# Adaptable, high recall, event extraction system with minimal configuration

**DOI:** 10.1186/1471-2105-16-S10-S7

**Published:** 2015-07-13

**Authors:** Makoto Miwa, Sophia Ananiadou

**Affiliations:** 1 The National Centre for Text Mining and School of Computer Science, Manchester Interdisciplinary Biocentre, University of Manchester, 131 Princess Street, Manchester, M1 7DN, UK; 2 Graduate School of Engineering, Toyota Technological Institute, 2-12-1 Hisakata, Tempaku-ku, Nagoya, 468-8511, Japan

## Abstract

**Background:**

Biomedical event extraction has been a major focus of biomedical natural language processing (BioNLP) research since the first BioNLP shared task was held in 2009. Accordingly, a large number of event extraction systems have been developed. Most such systems, however, have been developed for specific tasks and/or incorporated task specific settings, making their application to new corpora and tasks problematic without modification of the systems themselves. There is thus a need for event extraction systems that can achieve high levels of accuracy when applied to corpora in new domains, without the need for exhaustive tuning or modification, whilst retaining competitive levels of performance.

**Results:**

We have enhanced our state-of-the-art event extraction system, EventMine, to alleviate the need for task-specific tuning. Task-specific details are specified in a configuration file, while extensive task-specific parameter tuning is avoided through the integration of a weighting method, a covariate shift method, and their combination. The task-specific configuration and weighting method have been employed within the context of two different sub-tasks of BioNLP shared task 2013, i.e. Cancer Genetics (CG) and Pathway Curation (PC), removing the need to modify the system specifically for each task. With minimal task specific configuration and tuning, EventMine achieved the 1st place in the PC task, and 2nd in the CG, achieving the highest recall for both tasks. The system has been further enhanced following the shared task by incorporating the covariate shift method and entity generalisations based on the task definitions, leading to further performance improvements.

**Conclusions:**

We have shown that it is possible to apply a state-of-the-art event extraction system to new tasks with high levels of performance, without having to modify the system internally. Both covariate shift and weighting methods are useful in facilitating the production of high recall systems. These methods and their combination can adapt a model to the target data with no deep tuning and little manual configuration.

## Background

**Figure 1:**
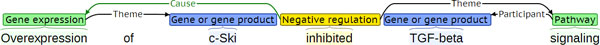
**Example of Event Structure in the CG task**.

Automatic extraction of biomedical events from texts has been a major focus of biomedical natural language processing (BioNLP) research in recent years. An event consists of a trigger expression (usually a verb or nominalisation), zero or more participants (arguments) of the events which may be entities or other events with their roles (e.g., *Theme, Cause*), and hedge attributes (e.g., *Negation, Speculation*). Figure 1 illustrates an example of event structures in the Cancer Genetics (CG) [1] task. In this figure, there are three events (*Gene_expression, Negative_Regulation*, and *Pathway*) that are represented with triggers (*Overexpression, inhibited*, and *signaling*), their roles (*Cause, Theme*, and *Participant*) and argument events or entities (e.g., *Gene_or_gene_products*). Several event extraction systems have been developed as a result of the BioNLP shared tasks (STs) since 2009 [2-4]. The systems have been applied both to PubMed abstracts and PubMed Central full papers. The extracted events have been successfully used in the creation of resources, e.g., [5,6] and in practical applications, e.g., for pathway curation and reconstruction (PathText [7]).

The use of these resources and applications has revealed that a wider range of event types and structures needs to be discovered by the event extraction systems than those addressed in the earlier BioNLP shared tasks, in order that the extracted events are able to cover as many as possible of the phenomena described in biomedical articles. The BioNLP Shared Task 2013 (BioNLP-ST 2013) introduced several tasks to address the problem. In particular, the CG and Pathway Curation (PC) [1] tasks defined a range of new entity and event types in the context of biomedical problems that had not previously been dealt with in the shared tasks. Given that both CG and PC tasks cover a greater number of bio-entity, role and event types than previous tasks, e.g., GENIA [3], event recognition becomes increasingly difficult since the systems need to extract correct event types and structures from a larger number of possible types and structures. Although both the representation and format of events are shared among many of the BioNLP subtasks, most event extraction systems participating in the BioNLP shared tasks have focussed only on a limited number of specific subtasks. This is largely due to the difficultly in applying a specific event extraction system to different tasks without carrying out considerable modifications to the system. It can thus be very costly and time-consuming to tune an event extraction system to deal with new event types. Accordingly, we have developed a novel method which allows an event extraction system to be applied to new tasks with competitive levels of performance, but without the onerous effort of development and tuning.

In this paper, we describe the integration of our novel method within our state-of-the-art event extraction system, EventMine [8], allowing it to be adapted to new tasks without internal modification and only minimal effort, without sacrificing performance [9]. EventMine has been enhanced with *configurability*, that allows it to be applied to new tasks with minimum manual effort and *adaptability*, that allows it to retain competitive levels of performance. Adaptation of the system to new tasks requires only that changes are made to a configuration file that is used to specify the task-specific information. In order to allow the system to adapt itself to new task specifications and achieve consistent performance, without the need for exhaustive tuning of the (hyper-)parameters of machine learning algorithms in EventMine, we have integrated a weighting method, a covariate shift method [10,11], and their combination into EventMine. The enhanced, adaptable version of EventMine has subsequently been applied to new tasks, i.e., the PC and CG tasks of BioNLP-ST 2013. The state-of-the-art performance achieved by EventMine on these tasks (1st and 2nd ranking, respectively) clearly demonstrates that the system can successfully be adapted to multiple new tasks through the specification of only minimal configuration information, and without the need for deep tuning.

## Method

In this section we firstly introduce EventMine, and then describe how it has been extended to facilitate more straightforward adaptation to new tasks. We conclude by describing how it has been applied for the BioNLP-ST subtasks of CG and PC.

### EventMine

EventMine [8] is an SVM-based pipeline event extraction system that has been applied to several biomedical event extraction tasks, and has achieved the top-ranked performance on several corpora [8,12], in comparison to other systems. EventMine consists of four modules: a trigger/entity detector, an argument detector, a multi-argument detector and a hedge detector. The trigger/entity detector enumerates the triggers/entities in the training data, finds words that match the head words (in their surface forms, base forms using parsers, or stems) of the triggers/entities, and classifies each word into specific entity types (e.g., *DNA_domain_or_region*), event types (*Regulation*) or a negative type that denotes that the word does not participate in any events. For example, the word *of *in Figure 1 has a negative type. The argument detector enumerates all possible event-role pairs among triggers and arguments that match the semantic type combinations of the pairs in the training data, and classifies each pair into specific *event role types *(e.g., *Binding:Theme-Gene_or_gene_product*) or negative role types (e.g., *Binding:NONE-Gene_or_gene_product*). In Figure 1, there is no relation that holds between *Overexpression *and TGF-beta, so they have a negative role type. Here, an event role type consists of a trigger type and an argument type with its role type. Similarly, the multi-argument detector enumerates all possible combinations of pairs that match the semantic type structures of the events in the training data, and classifies each combination into an *event structure type *(e.g., *Positive_regulation:Cause-Gene_or_gene_product:Theme-Phosphorylation*) or a negative type. Here, an event structure type consists of a trigger type and argument types with their role types. The hedge detector attaches hedge attributes to the detected events by classifying the events into specific hedge types (*Speculation *and *Negation*) or a negative type.

All the classifications are performed by using one-vs-rest support vector machines (SVMs). The detectors use the types or type combinations mentioned above as their classification labels. Labels with scores higher than the scores of the separating hyper-plane of SVM and labels with the highest scores are selected as the predicted labels. Classification is treated as a multi-class, multi-label classification problem with the requirement that at least one label (including a negative type) is selected during the prediction process. Classification makes use of both lexical and syntactic features. These features consist of character n-grams, word n-grams, shortest paths among event participants within parse trees, and word n-grams and shortest paths between event participants and triggers/entities outside of the events within parse trees. We replace all gold (given) entity names with their types to avoid the models being tuned to specific entities. To reduce feature space cost, we compress the feature space to 2^20 ^by hashing [13]. We assign greater weights to the positive instances to alleviate class imbalance and we normalise the feature vectors for each type (e.g., the word n-gram feature vector is normalised to a unit length) as well as for the entire vectors, and set the *C *parameter for SVM to 1.

EventMine generates training instances based only on predictions by the preceding modules in the pipeline, thus ensuring that training is not carried out on instances that cannot be detected by the preceding modules. If the generated instance corresponds to gold instances, then the semantic types assigned to the gold instances are assigned to the generated instance. Otherwise, a negative type is assigned to the generated instance. This mode of instance generation allows us to obtain similar distributions of training and test instances, as it is impossible to detect them if the participants are missed by the preceding modules.

### Extension of EventMine

This section describes how EventMine has been enhanced to allow it to be applied to new tasks with minimum manual effort, whilst retaining good levels of performance. We firstly explain the incorporation of a configuration file that allows EventMine to be applied to new tasks without internal modification of the system. Subsequently, we introduce three methods which, based on the information provided in the configuration file, allow EventMine to adapt itself to carry out new extraction tasks without the need for parameter tuning.

The TEES-2.1 system [14] has a similar motivation to ours regarding ease of adaptation to a range of different tasks, and it has been applied to several event extraction tasks in the BioNLP-ST 2013. Both TEES-2.1 and EventMine are pipeline-based systems and extract labels required for classification from the training data. However, they vary in terms of their approaches to implementing adaptability. TEES-2.1 does not require user-provided configuration information and applies an automated, but time-consuming, hyper-parameter tuning method that uses the development data set. In contrast, EventMine takes user-provided configuration information and employs three methods that remove the requirement for parameter tuning, as explained in the following section.

#### Configurability of EventMine

**Figure 2:**
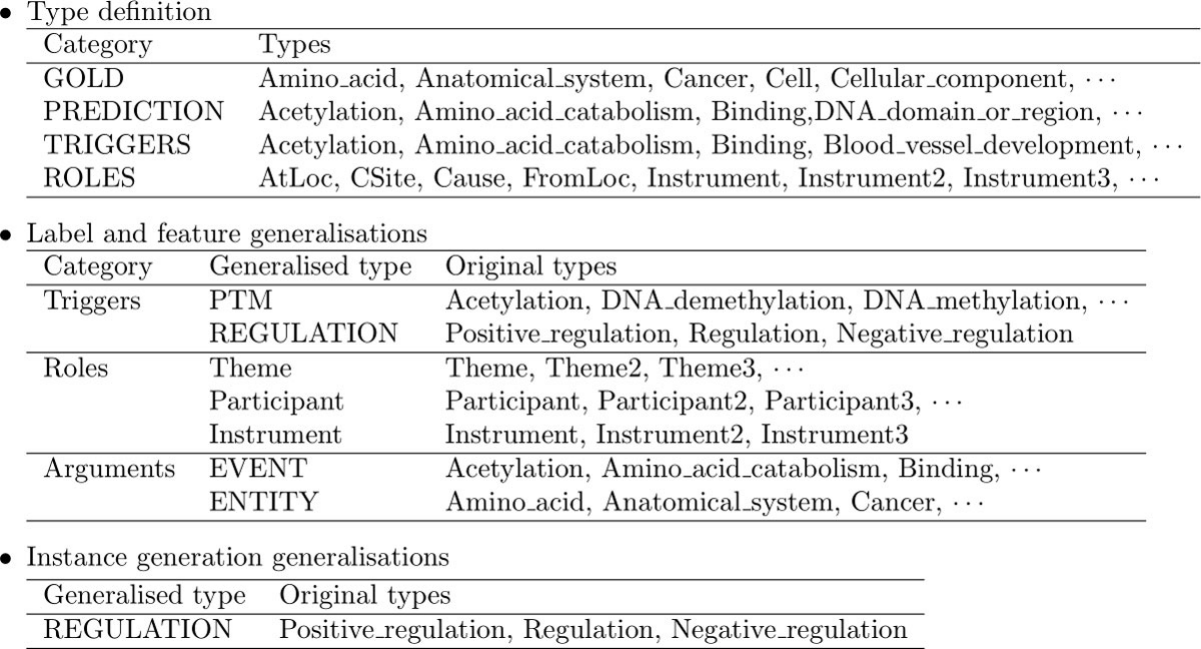
**Example of Configuration for the CG task**. Some types are omitted for brevity.

Configuration of EventMine to new tasks is facilitated through the specification of task-specific information within a configuration file. Figure 2 shows an example configuration for the CG task. This configuration file requires users to list the types of triggers (TRIGGERS in Figure 2) and roles (ROLES in Figure 2) of interest with listing gold trigger/entity types (GOLD in Figure 2) and trigger/entity types that need to be predicted (PREDICTION in Figure 2). Entities do not need to be explicitly specified under another "heading" in the configuration file because they can be calculated from the other "headings", i.e., entity types are those that are in GOLD and PREDICTION, but not in TRIGGERS. Based on the information provided in the file, EventMine enumerates all the structures among the user-specified types under both GOLD and PREDICTION that appear in the training data, and uses them to generate candidate instances for use in training each of the detectors in the pipeline introduced above. These type settings are also used so that EventMine can automatically enumerate gold triggers/entities in the training data and use them to generate candidate instances in the trigger/entity detector. These type settings are further required so that EventMine can be applied to annotated data without the need to prepare separate gold (*.a1) and prediction (*.a2) files, which are the official formats of the BioNLP STs. By specifying which types are gold in the configuration file, EventMine can flexibly change the task settings, e.g., the system can be configured to predict the types that are specified in the a1 files, or to treat types in the a2 files. Although this specification of types is indispensable for EventMine, it can be automated by creating the configuration file from the training data in the BioNLP ST format. Manual configuration is required only when users need to change the task settings and/or specify generalisations and other options, as detailed below.

The configuration file also includes two types of generalisations: one for *labels and features *("Label and feature generalisations" in Figure 2) and one for *instance generation *("Instance generation generalisations" in Figure 2). These generalisations are used in both training and prediction phases since they should be performed in similar situations.

*Label and feature generalisations *reduce the number of event role types and event structure types that are used as classification labels, and the number of features used by all detectors. The event role types and event structure types are combinations of types of triggers and participants with their roles, as explained in the *EventMine *section. The generalisations help to reduce the computational and space costs of both training and prediction since these are dependent on the number of the classification labels. The generalisations are indispensable for the two tasks in the BioNLP-ST 2013, since the tasks cover a greater number of bio-entity, role and event types than previous shared tasks, meaning that there are thousands of potential event structure labels. Considering all of these possible labels without carrying out generalisations would create an intractable problem for EventMine. Although the effects of the generalisations on event extraction performance cannot be evaluated on the tasks since it is infeasible to run EventMine without them, the generalisations have both advantages and disadvantages: the generalisations may alleviate the data sparseness problem during training, but they may also induce over-generalised features when they are applied to the tasks with enough training instances. The generalisations are applied to event role and event structure labels, since the types in these labels include types that are predicted by other detectors. For example, an event role label *Positive_regulation:Theme-Phosphorylation *contains *Positive_regulation *and *Phosphorylation*, which are predicted by the trigger/entity detector. Label and feature generalisations are possible in the following three cases: firstly, trigger/entity types are predicted by the trigger/entity detector, so their prediction is not required in the argument and multi-argument detectors. Secondly, the role types are predicted by the argument detector, so their prediction is not required in the multi-argument detector. Thirdly, the numbered role types, e.g., *Theme, Theme2*, are predicted in the multi-argument detector, so their prediction is not required in the argument detector. The numbered role types are required in events since the numbers indicate the correspondence between roles. For example, if an event has two *Theme*s and the second *Theme *has a corresponding *Instrument*, their roles will be *Theme2 *and *Instrument2 *to differentiate from the first *Theme *and *Instrument*. It is difficult to predict argument numbers without knowing the other arguments involved in the event, so the numbers are predicted in the multi-argument detector. These generalisations are also applied to the generation of the features used by all detectors. For example, generalisations of gold entities can be used as the basis of generating features used by the argument/trigger detector.

*Instance generation generalisations *are used to expand the possible event role types and event structure types to create instances in prediction. The instance generation generalisations may introduce noisy instances but they may also generate instances of event structures that otherwise would not have been considered, due to lack of evidence in the training data. For example, even if there are no *Positive_regulation:Theme-Gene_expression *instances in the training data, such instances are also created in prediction when there are *Regulation:Theme-Gene_expression *instances in the training data and there is a rule in the configuration file specifying that *Positive_regulation *and *Regulation *event types should share the event structures. The rules for the instance generation generalisations are applied whenever instances are created for prediction. The instance generation generalisations are included separately from the label and feature generalisations since the latter may introduce illegal or unrealistic event structures. For example, if we specify to share the event structures of *Phosphorylation *and *DNA_methylation *in instance generation in CG and transfer the event structures of *Phosphorylation *to *DNA_methylation, DNA_methylation *with *Molecule *as *Theme *will be illegally created (*DNA_methylation *takes only *Gene_or_gene_product *as *Theme *in the task definition.)

In addition to the task specific settings, the configuration file is designed to specify other options, e.g., parsers, domain adaptation methods, dictionaries, etc. Although we acknowledge that the achievement of high levels of performance on a specific task is largely dependent on determining the most appropriate combination of various methods and resources such as the above, our aim here is to demonstrate the configurability and adaptability of EventMine, rather than trying to achieve the highest possible performance for the tasks considered. Accordingly, the settings for the configuration options introduced above are the same as those used in our previous application of EventMine to the EPIgenetics and post translational modifications (EPI) task, as described in [8], unless otherwise noted. Specifically, we employ both a deep syntactic parser, Enju [15] and a dependency parser, GDep [16]. We utilise liblinear-java [17] with the L2-regularised L2-loss linear SVM setting for the SVM implementation, MurmurHash2 [18] for hashing, and Snowball [19] for stemming. We use no external resources (e.g., dictionaries) or tools (e.g., a coreference resolver) except for when we use external corpora to create stacked models for the PC task, as explained later.

#### Adaptability of EventMine

Although the above-described configuration file allows EventMine to be straightforwardly *configured *to new tasks, this does not in itself guarantee that the *performance *of the system on such new tasks will be of an acceptable quality. In other words, we need to ensure that EventMine is *adaptable *to new tasks. In all four modules of the event extraction pipeline, EventMine needs to solve classification problems. Some of the issues relating to the 1-vs-rest classification method employed are dependent of the settings of the hyper-parameters, which should be tuned to allow the classifiers to work to their full potential. However, it is costly and time-consuming to search for the best setting from the many possible hyper-parameter combinations. There is no general, efficient method to automatically tune the parameters within in a pipeline setting and it is also unrealistic to assume that the hyper-parameters can be effectively tuned for new tasks without exhaustive searching and knowledge of how the system works.

In order to ensure effective and efficient adaptability of EventMine, we have developed a novel adaptation process that avoids the need to carry out hyper-parameter tuning when the system is being configured for new tasks. This process makes of two different methods, i.e., a weighting method and a covariate shift method [11]. The latter is a novel approach, which has not been employed in any other event extraction system. We also combine the methods, and investigate the potential benefits of this combination. The weighting method adds weights to the positive instances in the training data. Although this method has previously been integrated into EventMine [8] and its application in the context of event extraction is not novel, we introduce in order to realise our goal of achieving maximum adaptability of the system, and in order to investigate its combination with the covariate shift method. Weighting positive examples has been empirically shown to improve several event extraction tasks, since it alleviates the class imbalance problem. This weighting method modifies the objective function of an L2-regularised L2-loss linear SVM as follows:

(1) $$\underset{\text{W}}{\text{arg min}}\;{\text{w}}^{\text{T}}\text{w}+\frac{n_{n}}{n_{p}}C\overset{n_{p}}{\underset{i}{\sum }}loss\left({\text{x}}_{p_{i}}\right)+C\overset{n_{n}}{\underset{i}{\sum }}loss\left({\text{x}}_{n_{i}}\right)$$

Here, **x*_p _***and **x*_n _***are the positive and negative instances, *n_n _*and *n_p _*are the numbers of negative and positive instances, and *loss *is a squared hinge loss that is defined as *loss*(**x*_i_***) = max(0,1 - *y**_i _wx_i_***)***^2^*. **Weighting is used in the BioNLP shared task evaluation (2013). This objective function makes the cost of the errors for all the positive instances close to one for all the negative instances, in order to encourage the classifiers to avoid prediction scores that are biased by class imbalances. The function also assigns greater penalties to rare instance errors. *n**_n _***is usually larger than *n**_p _***in the 1-vs-rest SVM, and this function achieves high recall for each module and consequently for the entire pipeline process. High recall is desirable for practical applications like semantic search, since such applications need to recognise as many events as possible. Achieving high recall in each individual module of the pipeline can additionally be advantageous, since it can increase the number of training instances available for training successive modules in the pipeline. Covariate shift methods generally aim to address the problem of varying distributions of instances between a training data set (on which a model is trained) and a target data set (to which the model is applied). Our method estimates instance distributions in the target data set by solving an additional binary classification problem between training and target data sets using a logistic regression classifier [11]. The additional classification problem treats training data sets as one class and target data sets as another class. Equation 2 shows the objective function of SVM with the covariate shift method.

(2) $$\underset{\text{W}}{\text{arg min}}\;{\text{w}}^{\text{T}}\text{w + }C_{CS}\overset{n}{\underset{i}{\sum }}l\left({\text{x}}_{i}\right)loss\left({\text{x}}_{i}\right),l\left({\text{x}}_{i}\right)=\frac{n_{train}}{n_{t\text{arg}et}}\cdot \frac{P_{t\text{arg}et}\left({\text{x}}_{i}\right)}{P_{train}\left({\text{x}}_{i}\right)},\;C_{CS}=\frac{n}{\overset{n}{\underset{i}{\sum }}l\left({\text{x}}_{i}\right)}C$$

Here, *p**_train_***(**x*_i_***) and *p**_target_***(**x*_i_***) are the outputs of the logistic regression classifier, and *n_train _*and *n_target _*are the numbers of training and target instances. This puts weights *l*(**x*_i_***) on all the training instances according to their likelihood of appearing in the target data set. *l*(**x*_i_***) represents the test-to-training ratio *p*(**x|θ**)*/p*(*x*|λ), where λ denotes the training distribution and *θ *denotes the test distribution, and it is known that the loss on the test distribution can be minimised by weighting the loss on the training distribution with the ratio [10]. In contrast to the originally proposed method in [11], our novel method introduces *Ccs *that keeps the balance between the regularisation and loss terms, since the *l(x_i_)* can suffer from overfitting of the training and target instances and the imbalance of the numbers of training and target instances. If we set all the weights *l(X_i_)* to 1, this is equal to the objective function of SVM. This objective function tries to make the distribution of the instances close to one for the target data set. This means that it encourages the classifiers to learn more about instances that seem to appear in the target data set.

The weighting and covariate shift methods are not exclusive and it is possible to combine them. To take advantage of the strengths of both methods, we also propose in a novel way to merge the two objective functions in a straightforward manner.

(3) $$\underset{\text{W}}{\text{arg min}}\;{\text{w}}^{\text{T}}\text{w + }\frac{\overset{n_{n}}{\underset{i}{\sum }}l\left({\text{x}}_{n_{i}}\right)}{\overset{n_{p}}{\underset{i}{\sum }}l\left({{\text{x}}_{p}}_{i}\right)}C_{CS}\overset{n_{p}}{\underset{i}{\sum }}l\left({{\text{x}}_{p}}_{i}\right)loss\left({{\text{x}}_{p}}_{i}\right)+C_{CS}\overset{n_{n}}{\underset{i}{\sum }}l\left({{\text{x}}_{n}}_{i}\right)loss\left({{\text{x}}_{n}}_{i}\right)$$

If we set all the weights l(⋅) to 1, this is equal to Equation 1.

The weighting and covariate shift methods are incorporated into EventMine by applying them to all the classifications in the four modules of the system. The hyper-parameter *C *is kept to 1 for all the experiments, as mentioned in the previous section. These methods may not necessarily produce the same levels performance that could be achieved by parameter tuning through exhaustive search. However, given the costly nature of such parameter tuning, as described above, our method makes the problem of adapting EventMine to new tasks much more feasible, whilst still allowing good levels of performance to be achieved. Since it is difficult to carry out exhaustive parameter tuning, it is not possible for us to compare the results of our novel methods with those that could be achieved through such tuning. Instead, we show in our experiments that incorporation of the new methods within EventMine can improve the performance of the system on both the PC and CG tasks of the BioNLP-ST 2013, to a level that is competitive with other systems that participated in these tasks.

### Configuration of EventMine for BioNLP-ST 2013 tasks

In the following sections, we describe EventMine's configuration for the CG and PC tasks, based on the notions of configurability and adaptability.

#### Configuration for the CG task

The Cancer Genetics (CG) task [1] aims to extract information from bio-processes related to the development and progression of cancer. The annotations in the training data were based on the Multi-Level Event Extraction (MLEE) corpus [20].

The configuration for our shared task submission used several label and feature generalisations, which are shown in Figure 2. For the event role types, generalisations for the trigger types, role types and argument types were applied as follows. In terms of the trigger types, we generalised the three regulation types, i.e., *Positive_regulation, Regulation *and *Negative_regulation *into a single *REGULATION *type, and post-transcriptional modification (PTM) types (e.g., *Acetylation, Phosphorylation*) into a single *PTM *type. In terms of role types, numbered role types were generalised as non-numbered role type (e.g., *Participant2→Participant*). In terms of argument types, event types were generalised as a single *EVENT *type and entity types were generalised as a single *ENTITY *type. These generalisations, except for the entity generalisations, are the combination of the generalisations used in the GENIA, EPI, and Infectious Diseases (ID) [4] annotated corpora of the BioNLP-ST 2011 [8]. For the event structure types, the same generalisations are applied, except for numbered role types, which are retained, since these are important in differentiating different types of event structures. Unlike other types, the numbered role types in events are not predicted by any other modules than the multi-argument detector as we explained in the *Configurability of EventMine *section.

Further experiments carried out after the shared task involved a more fine-grained classification of entities into three general types defined in the hierarchy of entity types defined for the CG task, i.e., *anatomical, pathological*, and *molecular*, instead of using a single *ENTITY *type, as in our shared task submission. In terms of instance generation generalisations, we applied them only to the regulation event types, to avoid introducing unexpected event structures.

#### Configuration for the PC task

The Pathway Curation (PC) task [1] aims to support the curation of bio-molecular pathway models, with the training texts selected to cover both signalling and metabolic pathways.

For our shared task submission for this task, we incorporated a stacking method [21], by training our models, using the same configuration as described above, on seven other available corpora: GENIA, EPI, ID, DNA methylation [22], Exhaustive PTM [23], mTOR [24] and CG. The stacking method uses the prediction scores of all the models as additional features in the detectors. Although some of these corpora may not be directly related to the PC task and the models trained on them can produce noisy features, we have used all the corpora, since stacking has been shown to improve performance [12,20]. Also in common with our work on the CG task, we have carried out further experiments after the shared task. The stacking method was not employed in this latter set of experiments, since our aim was to focus on the three methods introduced in the section on *Adaptability of EventMine*.

We employ the same type of generalisations as in the CG task described in the previous section, except for entity types. For our shared task submission, entity types were generalised to a single *ENTITY *type, similarly to our submission for the CG task. For our experiments that followed the shared task, a different type of entity generalisations to the one performed for the CG task was carried out, according to the different entity type definition for this task. The only type of entity generalisation we performed in the context of the PC task was to collapse the *Gene_or_gene_product *and *Complex *types into a single *PROTEIN *type. The other two entity types used in the corpus, i.e., *Simple chemical *and *Cellular component*, retained their original labels. The generalisation is based on the *reference resources *of the entity type definition [1].

## Results and Discussion

We have evaluated EventMine using the various configurations introduced in the previous sections. We firstly evaluated the system using settings employed for our shared task submission, which incorporated the use of the configuration file and the weighting method, but not the covariate shift method and task-specific entity generalisations. We compare our official results with those achieved by the best system that participated in the PC and CG tasks apart from EventMine, i.e., TEES-2.1 [14]. This evaluation is also presented in [9]. Subsequently, we evaluated the differences in performance that were obtained through the integration of the weighting method, the covariate shift method and their combination, together with the refined entity generalisation settings.

## Evaluations on instance generation generalisations and stacking

We evaluated the effect of applying instance generation generalisations and stacking to the PC development data set. The results are summarised in Table 1. For this evaluation, we used the same settings as those used in our shared task submission, i.e., we added weights to positive examples as in Equation 1 and we generalised all entity types into a single type. The scores were calculated using the evaluation script provided by the organisers with the official evaluation metrics (soft boundary and partial recursive matching). The generalisations improved recall at the slight expense of precision, and they slightly degraded the F-score. The generalisations were applied to the test set in our shared task submission, since slightly higher recall is favourable for practical applications like semantic search [7]. Whilst the use of the stacking method slightly improved performance, this improvement is not statistically significant (*p *= 0.14) using the approximate randomisation method [2,25].

**Table 1:** Effect of instance generation generalisations and the stacking method on the PC development data set.

Generation	Stacking	Recall	Precision	F-score (%)
×	×	42.87	47.72	45.16
✓	×	43.37	46.42	44.84
✓	✓	43.59	48.77	46.04

Instance generation generalisations (Generation) and the stacking method (Stacking) are applied to the PC development data set.

## Official scores for the shared task

Tables 2 and 3 show the official scores of EventMine when applied to the test data sets for the CG and PC tasks. Our system ranked second in the CG task and first in the PC task. There were six participants for the CG task and two participants for the PC task, and the scores of the top performing systems (TEES-2.1 [14], NCBI [26], and RelAgent [27]) are shown in Table 2, and the scores of the best system among the other participating systems (TEES-2.1) are shown for reference in Table 3. The performance of the third-ranked system is 5.71% lower than our system, in terms of F-score, as shown in Table 2. Therefore, we will focus on the comparison with TEES-2.1. Our system achieved the highest recall for both tasks, which is considered favourable, as mentioned above. This high recall is understandable, since we solved the problems as multi-label classification tasks in which the class imbalance problem was addressed through the application of the weighting method and the incorporation of instance generation generalisations. The performance of EventMine on both the CG and PC tasks (in terms of F-score) is slightly lower than that achieved by the system on the GENIA and ID tasks in the BioNLP-ST 2011 [8], but is comparable to the performance obtained on the EPI task. This may be partly because the GENIA and ID tasks deal with fewer event types than the other tasks.

**Table 2:** Official best and second best scores on the CG and PC tasks.

Task	System	Recall	Precision	F-Score (%)
CG	EventMine	**48.83**	55.82	52.09
	TEES-2.1	48.76	**64.17**	**55.41**
	NCBI	38.28	58.84	46.38
	RelAgent	41.73	49.58	45.32

PC	EventMine	**52.23**	53.48	**52.84**
	TEES-2.1	47.15	**55.78**	51.10

Highest scores are shown in bold.

**Table 3:** Recall / Precision / F-scores for event categories on the CG and PC tasks

Task	Category	EventMine			TEES-2.1		
		**Recall**	**Precision**	**F-Score**	**Recall**	**Precision**	**F-Score (%)**

CG	ANATOMY	69.43	73.28	71.31	73.11	81.79	**77.20**
	PATHOL	56.51	63.44	59.78	61.69	74.54	**67.51**
	MOLECUL	72.03	73.53	**72.77**	67.33	78.76	72.60
	GENERAL	48.74	58.26	**53.08**	44.72	62.68	52.20
	REGULAT	37.00	43.02	39.79	37.17	51.21	**43.08**
	PLANNED	40.05	40.98	**40.51**	34.78	45.51	39.43
	MOD	22.85	43.44	29.95	24.89	57.07	**34.66**

PC	SIMPLE	66.42	64.80	**65.60**	60.40	67.87	63.92
	NON-REG	69.07	62.69	**65.72**	61.16	65.74	63.37
	REGULAT	37.73	42.79	**40.10**	35.17	44.76	39.39
	MOD	23.56	34.65	28.05	22.41	40.00	**28.73**

Highest F-scores are shown in bold. We refer the reader to the papers of the tasks [1] for the details of the event categories.

Although EventMine did not achieve the best overall results in the CG task, we still consider that the performance level achieved is promising, given that we did not incorporate any external resources, and we did not carry out any tuning of parameters (e.g., *C *in SVM). A detailed comparison with TEES-2.1 shows that TEES-2.1 outperformed EventMine in the recognition of anatomical and pathological event categories, which constitute event types that have not been addressed in previous shared tasks. This indicates EventMine missed some of the novel structures introduced in these new event types. However, EventMine performed better than TEES-2.1 in the recognition of some other types of events involved in the task. The performance range of EventMine in recognising the various event types covered by the CG task is similar to the scores achieved by the system when applied to the MLEE corpus (52.34-53.43% F-Score [20]) although we cannot directly compare the results since the corpora are not completely same and the test sets are different. The ranges of the scores are around 60% to 70% F-score for non-nested events (e.g., *SIMPLE*), 40% for nested events (e.g., *REGULATION*) and 30% for modifications (e.g., *MOD*). This large range of scores may be caused by a cumulative combination of errors in predicting triggers, participants and modifications, since a similar spread of accuracy has been observed for previous tasks (e.g., GENIA, EPI, and ID results in [8]). Also, previous tasks like GENIA provided more training instances per type than the CG task, but the ranges of scores are broadly similar. These results indicate that further improvements to the performance of the system may require more than a simple increase in the training instances. EventMine performed particularly well on the PC task which is an encouraging result in demonstrating the adaptability of the enhanced system, since it was a completely novel task for the system. The recall achieved by our system was considerably higher than that obtained by TEES-2.1.

## Evaluations on the weighting and covariate shift methods

Our next set of experiments evaluated the weighting method, the covariate shift method, and their combination, explained in section on *Adaptability of EventMine *(see. Equations 1-3.) As a baseline system comparison, we used the version of EventMine that did not incorporate these methods.

As explained in the description of the task-specific configurations of the system above, this evaluation differed from the other evaluations in two ways. Firstly, stacking was not employed for the PC task. Secondly, we used refined entity type generalisations, i.e., detailed entity type generalisations based on the individual task definitions were employed in the task settings.

We show the results of the evaluations for the CG and PC development data sets in Table 4. Without the integration of the new methods, EventMine tends to produce high precision results, but with a large imbalance between precision and recall. The tendencies towards high precision are also seen in the results obtained by EventMine in the BioNLP-ST 2013 evaluation (see Table 2).

**Table 4:** Effect of the weighting and covariate shift methods on the development data sets.

Task	Weighting	Covariate shift	Recall	Precision	F-score (%)
CG	×	×	40.70	62.19	49.20
	✓	×	50.11	50.68	50.39
	×	✓	44.42	59.78	50.96
	✓	✓	48.83	54.10	51.33

PC	×	×	37.89	61.26	46.82
	✓	×	44.23	49.18	46.57
	×	✓	40.65	55.81	47.04
	✓	✓	42.73	52.13	46.97

As evidenced in Table 4, the use of both the weighting and covariate shift methods improve the recall by a significant margin. Additionally, the previously mentioned discrepancies between precision and recall become far less pronounced when these methods are employed. In most cases, a slight increase in F-score is obtained, with the exception of when the weighting method alone is applied to the PC development set. In terms of results achieved when the weighting and covariate shift methods are combined, the improvement observed on the CG development set is statistically significant, i.e., *p *= 0.014. This improvement is surprising, since the discrepancy of the distributions between the training and test data sets is considered to be small. This is because the shared task data sets are controlled - the documents in the training and development data sets are selected using the same criteria and the separation aims for an even distribution of instances between the training and development data sets. This improvement serves to demonstrate the difficulty in preparing controlled separation of data sets. The covariate shift method accesses the target data in advance and tunes the system for the target data, meaning that the model is not suitable for application to other data. Thus, it may not be appropriate to compare the results obtained on the test set with the results of other experiments. The improvement observed through the application of this method, however, provides evidence that it is possible refine the model based on the target data in a general way. This is important when we apply the models to other document collections, such as PubMed. The results in Table 4 demonstrate how the incorporation of the adaptation methods enhances the performance of the system on the PC development set, compared to the version of the system used for the shared task (Table 1). The comparison of these tables shows that exhaustive entity type generalisation (i.e., the removal of all semantic information about entities) had a negative effect on precision for the PC task, and that incorporating some simple task specific knowledge, by assigning generalised semantic types to the entities, according to the individual task descriptions, can boost the performance of the system. We also compared the baseline method with the combination of the weighting and covariate shift methods on the CG and PC test data sets, as shown in Table 5. Similarly to Table 4, the combination method improved the recall performance on both test data sets by a large margin in total (*TOTAL *in Table 5). As a result, the method improved the F-scores achieved by the system. For the CG test set, the use of the combined method shows a slight improvement over the use of either of the methods individually (when used with task-specific entity generalisations). For the PC task, a lower F-Score was obtained than for the original shared task results shown in Table 2. Since that version of the system made use of the stacking method, the positive effect of making use of information from multiple corpora is clearly demonstrated. Table 5 also shows that the combination method improves the recall performance on all the categories, and improves the F-scores except for *ANATOMY *(anatomical events) and *PATHOL *(pathological events) in the CG task and *NON-REG *(non-regulation events) in the PC task. This result indicates that the combination method does not work well on these categories, and this might be one of the reasons why EventMine cannot match the performance of TEES-2.1 on the *ANATOMY *and *PATHOL *categories. In particular, the method significantly improved the performance on *MOD *(modifications). This is partly because the high recall increased the number of training instances for modifications, and also partly because the method reduces the imbalance between precision and recall.

**Table 5:** Effect of the weighting (W) and covariate shift (CS) methods on the test data sets.

Task	Category	Recall	Precision	-W -CS F-Score	Recall	Precision	+W +CS F-Score (%)
CG	ANATOMY	70.17	80.39	74.93	74.37	72.76	73.56
	PATHOL	60.54	75.96	67.38	67.05	67.31	67.18
	MOLECUL	58.75	81.24	68.19	73.56	72.81	73.18
	GENERAL	38.69	65.25	48.58	47.24	52.37	49.67
	REGULAT	28.52	52.72	37.02	38.35	41.36	39.80
	PLANNED	33.41	51.41	40.50	46.45	40.20	43.10
	MOD	9.28	67.24	16.30	26.92	45.93	33.95
	TOTAL	41.88	67.18	51.59	51.96	54.77	53.33

PC	SIMPLE	58.52	76.78	66.42	66.53	68.05	67.28
	NON-REG	60.27	72.65	65.88	67.93	62.83	65.28
	REGULAT	25.94	53.69	34.98	36.28	43.34	39.49
	MOD	5.75	38.46	10.00	22.41	39.53	28.61
	TOTAL	41.62	65.44	50.88	50.93	54.10	52.47

Stacking is not used for the PC task.

## Conclusions

In this paper, we have described the development of an adaptable event extraction system, which accepts task-specific information in the form of a configuration file, and employs methods that alleviate the need to carry out extensive tuning of the system to allow it to be applied to new data sets. The new system has been created by enhancing an existing state-of-the-art event extraction system, EventMine. The configuration file is used to specify the definitions of types (e.g., entity and event types) and generalisations over these types that are used to adapt the system to new tasks. The provision of this configuration information alleviates the need to carry out task-specific modification of the system. Furthermore, to avoid the costly process of extensive parameter tuning to make the system suitable for application to new data sets, three adaptation methods are employed, i.e., a weighting method, a covariate shift method and their combination. The weighting method aims to alleviate class imbalance, while the covariate shift method aims to automatically adjust the differences in the distributions of instances in the training and target data sets. The enhanced system was applied to the CG and PC tasks with minimal task specific configuration. In the context of the BioNLP-ST 2013, only the weighting method was employed to facilitate the adaptation of the system to the specific tasks. This version of the system achieved the second best performance in the CG task and the best performance in the PC task. Following the shared task, we were able to further improve the results, though the incorporation of the covariate shift method, combined with task-based generalisation of entity types, the latter of which preserves some semantic information about entities that was lost in the more extreme generalisation of the entities used in the shared task version of the system.

The positive results obtained through the integration of our novel methods demonstrate that the enhanced version of EventMine can be effectively adapted to new tasks, without the need to make changes to the system itself. The weighting method, covariate shift method, and their combination have all been demonstrated to be useful in facilitating automatic tuning of the system to the new tasks. The success of applying the covariate shift method to the shared task data underlines its potential importance in future event extraction research, as a means to resolve the differences between the training and target data sets, which is a vital step to support the development of accurate and practical applications. Based on these results, our future work will involve investigating the feasibility of applying the covariate shift method to larger data sets e.g., PubMed, as a basis for the development of more practical applications. In this scenario, however, and in contrast to the training and test data sets of the shared tasks, there remain several issues to be resolved. These issues include the much larger differences in distributions between the training and target data sets, the vast number of target documents and the lack of standard evaluation criteria. Moreover, unlike in the shared tasks, the named entities are not given and their detection exhibits similar problems to the above. Whilst these problems are all challenging, we believe that it is vital for them to be addressed, in order to facilitate a significant improvement in the adaptability of event extraction systems to new tasks, and to allow their use in new practical applications.

## Competing interests

The authors declare that they have no competing interests.

## Authors' contributions

Both authors contributed to the production of the manuscript. MM built the system and carried out the experiments. SA supervised all steps of the work. Both authors read and approved the final manuscript.

## Declarations

The publication costs of this article were funded by the University of Manchester. This work was supported by the Medical Research Council [grant number MR/L01078X/1], the Arts and Humanities Research Council (AHRC) [grant number AH/L00982X/1] and the JSPS Grant-in-Aid for Young Scientists (B) [grant number 25730129].

This article has been published as part of *BMC Bioinformatics *Volume 16 Supplement 10, 2015: BioNLP Shared Task 2013: Part 1. The full contents of the supplement are available online at http://www.biomedcentral.com/bmcbioinformatics/supplements/16/S10.
